# “What I share is not the same as therapy”: Psychologist experiences of Instagram use as a mental health influencer

**DOI:** 10.1111/papt.12585

**Published:** 2025-02-21

**Authors:** Ella White, Terry Hanley

**Affiliations:** ^1^ University of Manchester Manchester UK

**Keywords:** ethical guidelines, mental health, psychologist, social justice, social media

## Abstract

**Introduction:**

Psychologists are increasingly using social media to share their therapeutic knowledge. Despite this, social media guidelines devised by professional bodies remain limited in content, with the focus typically on personal use rather than professional use as a mental health influencer. Therefore, this study aimed to explore practitioner psychologists' experiences of Instagram use with an influencer presence.

**Methods:**

Twelve UK‐based practitioner psychologists were interviewed who had an Instagram account that they used as a mental health influencer. The semi‐structured interview transcripts were analysed using reflexive thematic analysis.

**Results:**

Three themes were developed, each with four subthemes. The first theme ‘“I'm a psychologist, but I'm not your psychologist” explored psychologists’ experiences of trying to input boundaries on Instagram around self‐disclosure, risk management, time management, and client work. The second theme ‘“anxiety about not wanting to do the wrong thing” highlighted the impact of an influencer presence on psychologists’ own mental health. The third theme ‘“I share what I think is going to be helpful for other people and myself” considered the psychologists’ motivations for Instagram use for social justice advocacy and business promotion, and the contradictions between these.

**Conclusion:**

Psychologists can use their training to share evidence‐based research as a free widely accessible form of psychoeducation potentially acting as a first step into therapy. However, there are still many ethical ambiguities thus updated guidance specifically for an influencer presence can reduce psychologists' anxieties and potential risks.

## INTRODUCTION

A mental health influencer (MHI) is a qualified therapist, trainee therapist, or other mental health professional who shares psychoeducation and anecdotes from their professional or personal experiences (Triplett et al., 2022). The role of an influencer or content creator integrates a personal presence and business presence together to share psychoeducation and resources encouraging people to seek mental health support (Smith et al., 2021). An influencer presence requires psychologists to use authenticity and self‐disclosure, so there is overlap between the personal and the professional (Hund, 2023). Although some psychologists may not self‐identify as MHIs, they may meet this criteria due to their content shared.

Multiple opinion pieces provide recommendations for psychologists using social media for personal use, such as contracting with clients around boundaries for social media use (Baier, 2019; Campbell et al., 2016). Despite the transferability of some of these recommendations, others are incompatible with social media use as a MHI. For example, psychologists are recommended to hide their social media profiles from search engines and use pseudonyms, to increase the difficulty of being found by clients. However, MHIs aim for their profile to be visible and accessible to all. The studies also advised not posting personal information. Conversely, this may prevent psychologists from being able to increase knowledge of the role of a psychologist and limit how authentic psychologists can be without any self‐disclosure. Baier (2019) advise keeping online self‐disclosure to a minimum to reduce risk of harm to the therapeutic relationship if the client found the therapist's account. This creates an ethical dilemma for psychologists in considering that any information shared about themselves on social media now counts as self‐disclosure that may be viewed by past, present or future clients, and so the impact of these posts on clients' needs to be considered (Zur et al., 2009). The psychologist's own safety when deciding what to share on social media must be considered, as there are potential risks for psychologists around stalking and harassing behaviour (Kivisto et al., 2015). The BPS (2023) suggest holding separate personal and professional accounts, only sharing personal information on the professional account if it is relevant to work.

Within the UK, the HCPC (2024a) have published specific guidance on social media to accompany their revised standards of conduct, performance, and ethics (HCPC, 2024b). The document references that registrants must meet these standards when using both personal and professional accounts, thus directly acknowledging health influencers. Multiple standards around the maintenance of appropriate boundaries are applicable to social media use, including using appropriate methods of communication to provide care and other services related to their practice. This suggests the HCPC perceive a professional presence on social media as providing care, and thus anyone who interacts with the practitioner's social media account could be perceived as a service user. However, questions are raised for if a psychologist's duty to reduce the risk of harm extends to social media. If a client's social media indicated suicidal ideation, Diamond and Whalen (2019) recommend the psychologist discuss this social media search with the client. However, it is unclear what responsibility the psychologist holds if this is not their client. Way (2020) reported that followers of Dr. Nicole LePera (@the.holistic.psychologist) highly praised her posts claiming they had learned more from these posts than from paid therapy leading them to reduce or even stop therapy. This raises concerns as Instagram posts are not tailored to the individual's needs and suggests a parasocial relationship is valued over a therapeutic relationship. Whilst psychologists sharing resources via social media, podcasts and books may be beneficial for the public's mental health, it should be transparent that this is not a direct substitute for therapy.

The APA (2021) social media guidelines state that psychologists are responsible for the accuracy of any psychoeducation they share online. Therefore, to negate the risk of spreading misinformation, psychologists are advised to share evidence‐based information backed by citations and informed by their own training and experience. The BPS (2023) social media guidance recommend psychologists avoid poor practice which includes sharing posts containing factual inaccuracy, unfounded assertions, and misrepresentation or selective presentation of evidence. Concerns have been raised for if some MHIs are pathologising normal experiences, with incorrect use of therapy language, such as attachment issues, which may create confusion and distrust of psychologists (Lilienfeld, 2012; Rudra, 2023).

The APA (2021) guidelines state psychologists also hold responsibility for sharing truthful and accurate information about their training, experience, and qualifications. Smith et al. (2021) recommend psychologists be clear on social media about their training and what services they can offer. Cederberg (2017) noted there is increased competition for psychologists to market themselves on social media, and to distinguish themselves from other practitioners, such as coaches, thus psychologists may be tempted to aggrandise their credentials. The general public may not fully understand the role of a psychologist, and thus make assumptions about the psychologist's competencies perceiving any content as reliable (McCashin & Murphy, 2023). The BPS (2023) guidelines concede that psychologists may have a large audience who may interpret information differently dependent on their level of knowledge about psychology. Psychologists also need to have knowledge of how to use the social media platform itself, such as how to create posts and how sharing psychoeducation to an individual differs from sharing to an unlimited number online (Smith et al., 2021).

### Rationale and research question

Although there is a wealth of quantitative research and opinion pieces exploring the above ethical issues from *personal* use of social media by psychologists, limited literature is available on *professional* use of social media. The literature indicates that professional use of social media by psychologists raises some similar ethical issues to personal use with other distinct issues also present, such as around competency (White & Hanley, 2022). Professional bodies have begun to publish social media guidelines with the brief inclusion of professional use of social media (APA, 2021; BACP, 2023a; BPS, 2023; HCPC, 2024a). This indicates recognition of the growing importance of this guidance. Despite this, more research is needed on different ethical issues that may be encountered dependent on the type of social media presence (personal versus professional), and how these issues can be overcome or avoided.

Currently, there is no qualitative literature exploring psychologists perspectives of holding a professional presence on social media. Therefore, this study employed a qualitative design to interview psychologists who hold an Instagram account as a MHI. Based on this rationale, the following research question was explored: what are practitioner psychologists' experiences of Instagram use as a MHI?

## METHODOLOGY

### Design and procedure

An inductive qualitative research design was chosen as it is hermeneutic and phenomenological based on the interviews to centre the perspectives and experiences of the participants, rather than a deductive theory‐driven approach (Braun & Clarke, 2013; Howitt, 2019).

Reflexive thematic analysis was used, with a ‘Big Q' approach prizing subjectivity over ‘small q' values of replicability and reliability (Clarke & Braun, 2018; Kidder & Fine, 1987). This research is situated within a social constructionist epistemological stance, with the belief that knowledge is constructed within a social and cultural context, with meaning attached subject to change (Burr, 2015). From a relativist ontological stance, qualitative approaches provide the opportunity to understand how psychologists interpret their own reality as a MHI (Baghramian, 2019).

This study was reviewed at department level due to low risk and received full ethical approval from the University of Manchester. The project adhered to the ethical requirements of the HCPC (2016) and BPS (2021).

After expressing interest, participants were emailed the participant information sheet and consent form to sign before the interview. The interviews were conducted remotely via Zoom to remove accessibility barriers as participants were located in multiple areas of the UK and had minimal time available for participation alongside their work (Afzalan & Muller, 2018; Deakin & Wakefield, 2013).

Individual semi‐structured interviews were used, due to their flexibility to explore new ideas raised by participants, whilst still retaining consistency through the interview schedule (Braun & Clarke, 2006). The interview schedule (Appendix A) comprised 11 open questions to elicit rich data through as personal a response as possible (Willig & Rogers, 2017). The interviews lasted 30–80 minutes, with the average interview time 65 minutes. A debrief sheet was emailed detailing available support services and the rationale.

### Participants

The inclusion criteria are UK‐based qualified or trainee practitioner psychologists with a minimum of 1000 Instagram followers, classifying as a nano influencer (Henderson, 2020). Participants were recruited through purposive homogenous sampling, regarded as an effective sampling method to recruit available participants from a target population (Patton, 2002). Psychologists were direct messaged on Instagram from an account created for this purpose using the same initial recruitment message for all prospective participants. Participants were found through typing ‘psychologist’ into the search function of Instagram, and reviewing the followers of psychologist's profiles and comments sections for further psychologists to contact. One participant was recruited through snowball sampling requesting participants share the study with peers (Goodman, 1961).

Twelve participants were interviewed over a three‐month period from October 2022 to January 2023. This fits with Braun and Clarke (2016) reasoning that larger sample sizes risk missing construction of diversity and nuance. Meaning is generated through data interpretation and so sample size and data saturation cannot be pre‐determined (Braun & Clarke, 2021c). Table 1 details participant demographic information, with 75% of participants clinical psychologists and only one participant identifying as male (8%).

**TABLE 1:** Participant demographic information.

Participant Pseudonym	Age	Gender	Ethnicity	Profession	Service
Bella	41	Female	White British	Clinical psychologist	Private practice
Echo	45	Female	White British	Clinical psychologist	Third sector
Evie	36	Female	White British	Clinical psychologist	Private practice
Grace	41	Female	White British	Clinical psychologist	Private practice
Ivy	35	Female	White British	Clinical psychologist	Third sector
Laura	36	Female	White British	Clinical psychologist	NHS
Lily	34	Female	White British	Counselling psychologist	Private practice
Liz	43	Female	White British	Clinical psychologist	Private practice
Louise	45	Female	White British	Clinical psychologist	Private practice
Mia	41	Female	Mixeds background	Clinical psychologist	Private practice
Mitch	38	Male	White British	Clinical and forensic psychologist	NHS and HMP
Sophia	29	Female	White Asian	Sport and exercise psychologist	Private practice

Participants were asked if they would like to choose their own pseudonym. This acknowledges the inevitable power dynamics between researcher and participant and attempts to increase collaboration (Dill & Kohlman, 2014; Mertens, 2015). Many participants commented that the ability to choose their own pseudonym felt novel and unexpected.

### Analysis

The six recursive phases of reflexive thematic analysis outlined by Braun and Clarke (2021a) were used. The primary researcher (Dr Ella White) familiarised themselves with the data through conducting and manually transcribing the interviews. White systematically read each transcript highlighting sections and adding “pithy” labels to summarise key points that may answer the research question, generating 176 codes (Clarke & Braun, 2013). Semantic coding was utilised based on the surface meaning of the data to present the content as communicated by participants (Byrne, 2022). Key words were used from participants where possible when naming codes. Codes that shared a similar underlying concept were collapsed into the same overarching code, generating 19 final codes. The collapsed codes were configured into a concept map for clarity of the relationship between codes, then organised into candidate themes using mind maps. The maps illustrated the high level of interrelation between the codes/themes (Appendix B). Theme development and candidate themes were reviewed with their supervisor (second author) to check they made sense in relation to the codes, original transcript, and research question. The researcher considered the story of each theme to create illuminating names drawing upon “punchy” extracts of participant quotes (Braun & Clarke, 2021b; Clarke & Braun, 2013). Braun and Clarke (2021a) outline writing a brief synopsis of candidate themes which were emailed to participants to provide member reflections. Feedback was applied through a recursive process, which included changing some of the subtheme names and adding more to the story of positive experiences of social media use following reflections on the heaviness and negativity of the themes.

### Trustworthiness and reflexivity

This research was conducted in line with guidelines for qualitative research provided by Braun and Clarke's (2006) 15‐point thematic analysis checklist. The research was grounded in examples to illustrate the RTA and the primary researcher's subsequent understanding, including transcript excerpts. The use of NVivo 12 increases the reputability of this research, with an audit trail of each version of the codes and themes generated. Member reflections were conducted to provide credibility checks and reduce the subjectivity of the analysis (Braun & Clarke, 2013; Kornbluh, 2015).

Elliott et al. (1999) list owning one's own perspective as a key guideline for qualitative research. The primary researcher is an active Instagram user with a private personal Instagram account. They have viewed a large number of posts from MHIs which inspired this study as they questioned the ethics of the growing use of social media by practitioners and felt this was a gap in their training. They would be open to use of social media as a qualified psychologist provided they felt there was sufficient ethical guidance and professional and peer support. Their personal experiences may have provided them with biases around practitioner Instagram use which they strived to make explicit within a reflexive journal during data collection and analysis. The researcher was aware of their biases as a counselling psychologist, reflected in their choice to include a subtheme on social justice which is a core value of the counselling psychology identity (Winter, 2019). The second author is a HCPC registered Counselling Psychologist interested in the interface between therapy and technology.

## RESULTS

**FIGURE 1:**
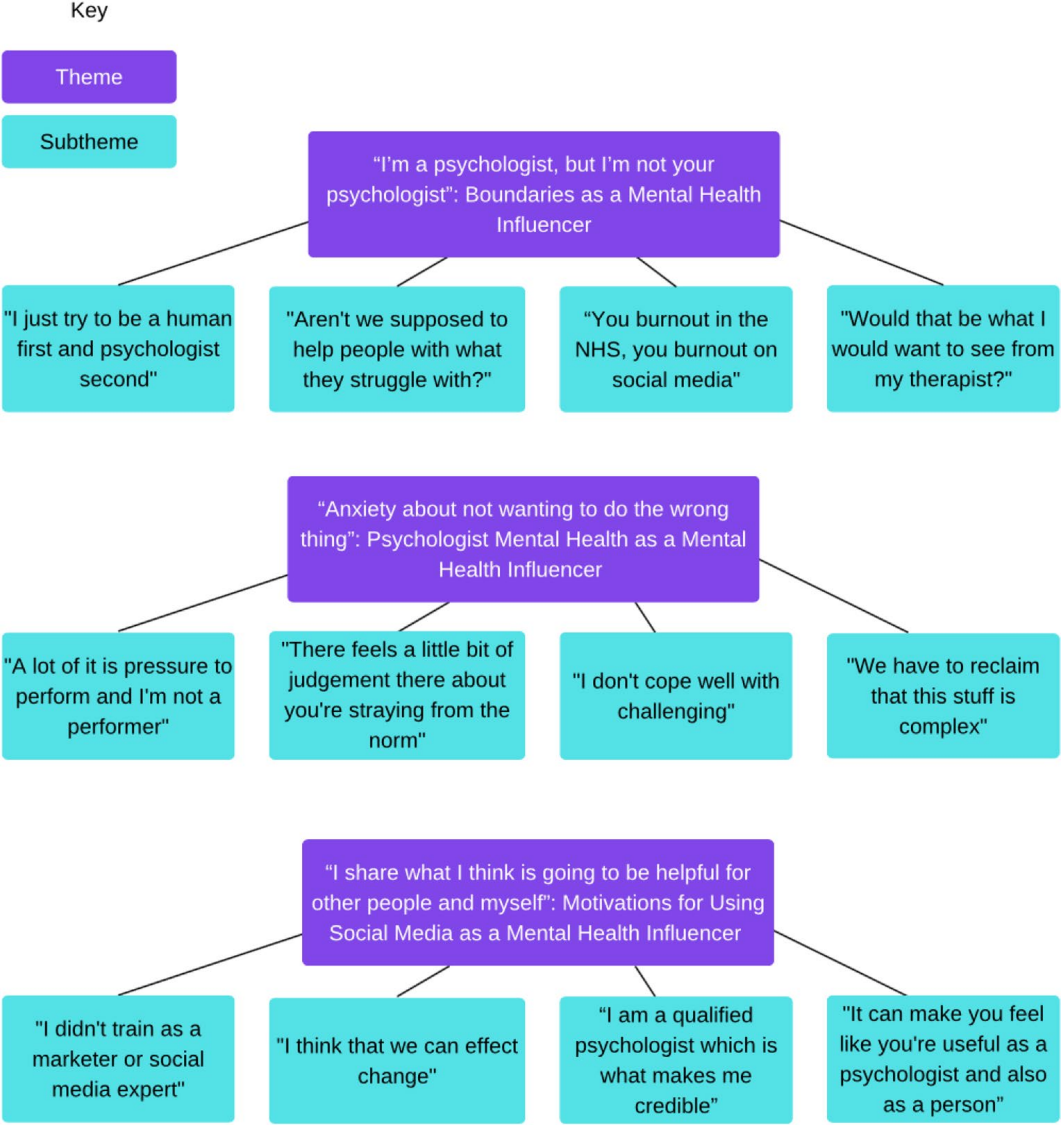
Final themes and subthemes.

Three themes were developed, each with four subthemes as visually illustrated in Figure 1. All of the themes and subthemes have been named using direct quotes from the psychologists in order to centre the psychologists' experiences, indicated by quotation marks.

### Theme one: “I'm a psychologist, but I'm not your psychologist”: boundaries as a mental health influencer

This theme is comprised of four subthemes and describes the issues that psychologists experience trying to input boundaries as a MHI.

“I just try to be a human first and psychologist second”

This subtheme describes psychologists experiences of balancing self‐disclosure with keeping themselves safe online. The psychologists believed it was important to use authenticity to show their followers they are also humans experiencing their own issues. Bella described this as a *“human first and psychologist second”* approach. Echo described the purpose of her self‐disclosure to *“make the material more tangible and easier to connect with”*, thus self‐disclosure serves a dual purpose to normalise psychological distress and increase relatability.

Sophia questioned if authenticity and informality actually created a *“false relaxedness”* as potential clients would be *“quite shocked then because it's suddenly got formalities”* around contracting and boundaries within therapy. Grace felt an *“overwhelming”* parasocial relationship *“because they think they know you […] so then some people may become really preoccupied with wanting this idealised version of you as their therapist*” with these high expectations likely different from the actual therapist. Therefore, whilst this authentic self‐presentation online is intended to strengthen potential therapeutic relationships, it may have the opposite effect.

Some psychologists felt they shared the same online as with their clients, whilst Lily acknowledged “*I probably wouldn't share all that information with one person in therapy because of the context”*. The psychologists were cautious around self‐disclosure that could risk their safety, with Echo stating, *“I'm very careful not to talk about where I live or to do posts with images of things in the background that are identifiable”*. Most psychologists disclosed if they had children; however, most did not share their children's faces or names as their children could not consent and they believed it unlikely to bring more work or connection to their followers.

“Aren't we supposed to help people with what they struggle with?”

This subtheme considers how psychologists input boundaries around supporting people online. Lily succinctly stated, *“I always make it really clear, you know, that I'm a psychologist but I'm not your psychologist”*. Moreover, Louise stressed that *“what I share is not the same as therapy, it's for the purposes of education”*. Many psychologists used an Instagram highlight disclaimer to explicitly state the purpose of their account and encourage people to seek offline individualised support with signposting to crisis services.

However, many psychologists felt a caring responsibility to validate distress without crossing into a therapeutic relationship. Ivy detailed her difficulties with responding to distressed messages as *“I don't want people to feel unheard”*, opting to reply but *“curtail it quite quickly which is not always easy”*. Mitch reflected that *“it's a bit rubbish to kind of not be able to help somebody […] it feels a little bit invalidating”*. An inability to support people online appeared to conflict with the psychologists values, with Sophia questioning *“aren't we supposed to help people with what they struggle with?”*. This is further contextualised by Echo acknowledging *“I certainly don't know the background and the context”* and Lily noting *“they're not often in the same country as you”* so could only provide generic advice in response to requests for support. Grace reflected on the ethics of posting potentially triggering content, particularly regarding trauma, and the importance of setting aside time for containment and signposting as “*I think that we have a duty of care to really not just dump stuff and run”*. Contrastingly, Echo believed that although *“you would be careful about how and what you shared”* it was the individual's responsibility to seek support if needed as *“I'm not clinically responsible for people that aren't my clients”*.

“You burnout in the NHS, you burnout on social media”

This subtheme details the burnout experienced by psychologists from being constantly accessible online. Sophia described feeling *“vulnerable”* in terms of the content, timing and quantity of messages (Ivy reported *“I get hundreds of messages a week*”). Sophia explained *“you are really putting it over to the other person to decide where and when and what they're going to be saying to you”*. The psychologists were concerned about burnout from messages containing risk or trauma, as Grace described *“you might not be ready or prepared”*. Laura described a follower disclosing *“they were feeling quite suicidal then that really affected me for a couple of days”*.

The psychologists were also concerned about burnout from balancing their Instagram with other work, with Mia experiencing a *“burnout cycle”* echoed by Liz's experience of *“a real drain on my energy, but there are definitely times when I feel like I get a real buzz”*. The psychologists reported issues with time management and tiredness making content whilst also having other work and family. Liz reflected *“I love the content creation, I just don't like how long it takes me”*, with Mitch adding *“whilst this is a hobby, it does take quite a lot of intellectual capacity and cognitive load”*.

“Would that be what I would want to see from my therapist?”

This subtheme is focused on managing boundaries around social media with clients. Whilst some psychologists did not disclose their Instagram presence to clients, others spoke in depth about their contracting process. Louise encouraged clients not to interact at all with her online *“because it could jeopardise their confidentiality and their privacy”*.

Some psychologists gained inspiration for content from their client work, either from a specific session or a more general theme. Sophia worried *“there's a possibility that they may think that some of the challenges they are having is content which is an ethical dilemma”* which could negatively impact the therapeutic relationship. Ivy kept her content unrelated to her client work as she empathised with her clients concern of *“is that post about me?”*

The psychologists held in mind their clients when making content, with Lily questioning *“if any of my clients saw this how would it impact them?”*. Sophia worried how the therapeutic relationship would be impacted if she behaved differently on Instagram compared to in 1:1 work, such as posting reels with music. Sophia asked herself *“how would I feel with potential clients seeing that? Would that help or harm that relationship? Would that be what I would want to see from my therapist?”*. Mia warned clients if she planned to post something that may be triggering or conversely very light‐hearted whilst they were going through a difficult time, exploring “*how it feels to see me show up in a different context when they feel like they need me to hold them in mind”*. This suggests Instagram may negatively impact the therapeutic relationship if the client sees the therapist outside of the therapeutic context.

### Theme two: “Anxiety about not wanting to do the wrong thing”: psychologist mental health as a mental health influencer

This theme centres around psychologists' mental health as a MHI and is constituted of four subthemes.

“A lot of it is pressure to perform and I'm not a performer”

This subtheme is focused on the felt pressure to conform to Instagram algorithms for engagement with posts. Evie reported *“a lot of it is pressure to perform and I'm not a performer”*, backed by Sophia recounting her experience of uploading reels despite feeling uncomfortable in front of the camera. This pressure to *“perform”* appears detrimental to the psychologist's mental health, and a potential deterrent for sharing on Instagram at all. This may become an increasing issue as Instagram's format has shifted to short videos. For Grace, the *“competitive”* algorithm has reduced her posting as *“what it's done for me is I've just withdrawn”*. The psychologists described pressure to keep up with changes to the algorithm, with not enough time to keep up with the changes.

Furthermore, many psychologists described feeling pressured to post regularly otherwise risk becoming in Sophia's words *“obsolete”*. The psychologists described their doubt and worry about the repercussions of posting less regularly with less engagement with posts and less work opportunities ranging from podcasts to client referrals. This anxiety also seeped into other work with Sophia explaining *“I think it was starting to impact my offline life because I was worrying about I need to get a post out which was distracting from the work that I needed to do.”* Therefore, risk of burnout is compounded by a pressure to post. The concerns described in the previous subtheme of how burnout could impact clients is heightened by Instagram also distracting the psychologist whilst at work.

“There feels a little bit of judgement there about you're straying from the norm”

This subtheme discusses the perceived judgement from other psychologists for using Instagram. Bella described social media work as *“a bit of a taboo […] some people don't like that you can earn money from anything other than one to one.”*. This is supported by Liz describing *“there feels a little bit of judgement there about you're straying from the norm”*.

Laura felt constricted in what she could post as *“I've had my employer ask me to delete certain posts because they haven't felt appropriate”* with confusion around this subjectivity. For Laura, this led to a *“hesitation putting certain stuff”* as *“that anxiety does come up from time to time as to how is this going to be received?”*. Some psychologists described NHS services having social media policies stipulating not posting political content, yet Evie continued to share social justice content as “*I'm staying true to my values and my beliefs, and if I lost my job then I lost my job”*.

Psychologists were also concerned with being reported to their professional body. Sophia felt *“paranoia”* and *“scrutiny from other practitioners about whether we should or shouldn't be doing it”* believing *“if anyone was to report me [to the HCPC] it'll be my fellow, it'll be my friend”*. Whilst all the psychologists reported a sense of community and a support network with other MHIs, this suggests an ‘us and them’ sense of paranoia being observed by psychologists without a platform.

“I don't cope well with challenging”

This subtheme explores psychologist experiences of criticism online. The psychologists attempted to minimise criticism online through supporting their content with evidence‐based research. However, many psychologists still were concerned with how their posts may be received, with Evie explaining that *“I do worry about it”* as *“there is that hesitancy, and that need to want to be able to back up whatever you said, and that fear of kind of recourse or people challenging what you say”*.

Multiple psychologists received criticism which they found difficult to manage. Ivy reported struggling with negative feedback to the extent that *“I can lose sleep over that actually”*. The negative feedback ranged from critique of the specific content they posted, to critique of the type of content they posted with Ivy angered by comments that social justice work is a psychologist's role. Mitch described how he had also experienced *“emotionally draining*” criticism of himself: *“sometimes your character can be challenged, sometimes your competency or knowledge can be challenged”*.

Some psychologists also reported self‐criticism, feeling either they were not good enough or their content was not good enough. Liz described choosing not to post as *“I'm a bit of a perfectionist and so, you know, I'll make a video and be like ‘oh the camera was wonky’ or ‘I stumbled on my words’”*. Bella described how *“sometimes the old impostor syndrome can slip in and a bit of jealousy as well”* with comparisons to other ‘successful’ psychologists online with more polished content, larger followings, and book deals.

“We have to reclaim that this stuff is complex”

This subtheme considers psychologists' frustrations with the oversimplistic format of Instagram removing the nuance from mental health content, termed by Mitch as a *“reductionistic approach”*. Ivy acknowledged the limitations of sharing drawings as *“you lose the nuance, and that's often the criticism I get actually is people are like well this doesn't apply to me”*. This suggests whilst the posts are intended to validate experiences, they can have the opposite effect where people feel invalidated that their experience differs from these short, oversimplified descriptions. Mia reflected that psychologists typically focused more on anxiety and depression as *“more palatable signs of mental health*”, rather than suicide and chronic mental health conditions, creating a *“glamorisation of mental health*” and thus potentially contributing to mental health stigma for what can and cannot be discussed.

The psychologists worried their content could lead to pathologisation of normal experiences, and cause self‐misdiagnosis rather than seeking professional support which contrasts with their intended purpose. This suggests an element of guilt, with Lily recognising she cannot control who views her posts or how they are received as “*this isn't intended for your average 15 year old but if they see it I don't want them to diagnose themselves or think there's something necessarily massively wrong with them”*. Many psychologists felt responsible for how their posts would be interpreted and did not want to create further distress. Mia described feeling *“very uncomfortable”* seeing *“quick fix stuff”* teaching coping strategies, fearing potential self‐blame as *“where people end up is well I must be broken then because I did all the things that they said to do and I still feel like shit”*.

### Theme three: ‘I share what I think is going to be helpful for other people and myself”: motivations for using social media as a mental health influencer

This theme, comprised of four subthemes, is focused on what the psychologists feel their purpose is for using social media as a MHI.

“I didn't train as a marketer or social media expert”

This subtheme explores psychologist views of using Instagram as a business, with Grace acknowledging *“you should be compensated for the work that you do since Instagram is a really hard job”*. Bella explained her stepped approach, with some free content followed by passive products behind a paywall, explaining “*everybody who's doing really well […] have passive stuff that they drive people to”*. Questions were raised on the relationship between the quality of content and the cost, with Sophia querying *“what's the difference between the stuff that I will put behind a paywall and like the quality of that information?”*. This was particularly relevant in the present socioeconomic context, with the power dynamic of a psychologist encouraging people to pay for support during the cost of living crisis.

Multiple psychologists used Instagram to gain clients for their private practice, with Liz recognising “I *definitely think it's a huge tool in me getting work.”* Evie commented *“[I'm] very transparent on social media. This is who I am so that the person knows what they're coming for before they start, because private work is really expensive and you want to get a good fit straight away ideally”*. This suggests Instagram benefits clients to learn about therapists to make an informed choice beginning therapy.

Many psychologists questioned the incompatibility of general marketing guidance to promote services with the ethical standards of a psychologist, with Grace describing how *“promoting yourself feels really icky”*. This ethical incompatibility felt particularly resonant in Mitch's concerns around Instagram as a tool for *“leveraging people's pain”* and *“offering them a solution for a price”* such as buying their book. Whilst some psychologists felt pride in developing their marketing skills, others felt uncertainty primarily due to a lack of training. Liz outsourced her marketing so she could focus on what she does have the psychology skillset for, explaining “*I'm not a marketer, I'm a therapist”*. This highlights a misconception that only one person runs the account, feeding into burnout and imposter syndrome for psychologists who do not employ people for these purposes. This also casts doubt on the importance of authenticity when the psychologist may not create all of their content.

“I think that we can effect change”

This subtheme regards using Instagram with a social justice agenda. Increasing accessibility to psychoeducation was a key reason for many psychologists to use Instagram, with Mitch recognising *“it needs to be about sort of reducing the stigma around mental health needs, it needs to be making information more accessible.”* Evie conceded Instagram facilitated her knowledge to be *“shared more globally”* to support more people than possible through individual therapy.

This was contextualised by long NHS waiting lists, with Mia describing how *“it brings up a lot of feelings of rage” and “I definitely felt much more defeated this year”* being unable to quickly refer people to support services. This helplessness acted as Mia's motivation to use Instagram *“as a space to share […] what's going on politically and financially and the impact that has on mental health”*. Mia aimed to help people realise *“I thought this was my problem and now I realise that it's a systemic problem”*. Multiple psychologists recognised they could link systemic issues to mental health due to their professional training and felt compelled to share this knowledge with the public to reduce self‐blame. Ivy felt empowered sharing systemic issues in line with her social justice values, explaining “*I think one way of affecting change is by kind of maybe highlighting some of the issues, and getting us to zoom out and think about the wider context”*.

A few psychologists referenced their guilt at leaving the NHS for private practice, using Instagram to still support a more diverse range of the population without the cost barrier. However, despite some psychologists naming social justice advocacy as the primary purpose for their account, the psychologist still benefitted from the posts attracting people to their private practice or passive products if offered. Lily reflected on how many therapy sessions began with psychoeducation and so providing these resources can act as a first step before beginning therapy as *“if they've already got that, that can make a difference.”*

“I am a qualified psychologist which is what makes me credible”

This subtheme considers the responsibility some psychologists feel to use their professional knowledge to share credible psychoeducation, as Grace reflected *“if we don't then there isn't going to be good information out there”*.

Laura acknowledged the power of the *“symbolic”* doctor title as “*it suggests a certain level of training and competency and trust”*. Evie explained how *“education is a validation in our culture”* so *“my voice is more likely to be heard and believed”*. This suggests psychologists believe including their doctor title in their Instagram handle validates the credibility of their content. Many psychologists felt pressured to market themselves as an expert, with a few tempted to step outside of their competencies for job opportunities as Mitch reflected *“I don't think that people really understand fully what a psychologist does”*. Bella reflected on the importance of psychologists staying within their competency as due to the power of their title “*people will not look into your qualifications and will assume that you are qualified”*.

The psychologists appeared conflicted in their views of the public sharing their lived experiences, as they could not provide the nuance and evidence base deemed highly important to not be invalidating. However, Evie questioned *“who defines what's accurate, what's truthful, what's reality?”* to validate that people without the privilege of mental health training still had a right to share their experiences. The psychologists were concerned about other health professionals discussing mental health, as they had the power of a professional status without mental health training. These experiences of viewing others sharing psychoeducation appeared to heighten the psychologists' responsibility to stay online, whilst adding concern their voice may be lost amongst many.

“It can make you feel like you're useful as a psychologist and also as a person”

This subtheme considers how the psychologist may benefit as a MHI. Whilst much focus has revolved around ethical issues, it is important to highlight psychologists enjoyed using Instagram with Lily explaining *“in some ways like this doesn't feel like work, it's like a hobby and so I quite enjoy doing it”*. Laura explained she could use her content for multiple purposes, so her staff team could benefit from her creatively presenting content which she could then upload to Instagram. Almost every psychologist referenced the relationships they had built, with Laura describing how *“there's been a real sense of community in joining Instagram which I wasn't expecting”*. Liz described how Instagram has reduced her sense of isolation working remotely through interacting with other psychologists on Instagram who have “*become each other's cheerleaders”*.

Furthering this sense of community, some psychologists created informal peer support groups with other psychologists on Instagram. Grace described using the group *“to pull each other up on a few things or ask for advice so I would say that was really invaluable”*. Most psychologists did not discuss Instagram in supervision, so peer support facilitated managing ethical issues. This experience‐informed support may be more effective as many supervisors are unfamiliar with ethical issues as a MHI.

Echo described how Instagram could be a *“double‐edged sword”*, contrasting *“nice validation […] and feeling that you're useful”* with the *“dopamine hit when you get a like so I'm sure it does in an unhealthy way impact me”*. Grace described how *“I take my time when I write posts and really consider who is this for?”* to reduce the risk of posting for an ego boost.

Lily described her confusion around the motive for psychologists to use emotional self‐disclosure, such as sharing images of themselves crying. Echo reported using examples of regulating her emotions on Instagram to normalise these experiences for her followers. However, she learned where to set the boundary as *“I wouldn't become like completely dysregulated and talk about my relationship failures there, because I don't think that would be helpful”*. Sophia wondered how clients would respond to seeing her upset online as *“now that person knows that I was sad on Tuesday”*.

## DISCUSSION

This study aimed to explore practitioner psychologists' experiences of Instagram use as a MHI. Despite psychologists using Instagram to try to support the general public with their mental health, these accounts appeared to have a variety of detrimental effects on the psychologists' own mental health. The psychologists shared their experiences of perceived judgement from other health professionals for using Instagram as this strayed from the ‘traditional’ psychology career. This feeling of judgement increased the psychologists' fear of being reported for misconduct online and prevented seeking support from others. Some of the psychologists described the criticism they had received in response to their posts, creating feelings of anxiety and loss of sleep, with other psychologists experiencing more serious trolling. The psychologists also described self‐criticism and self‐comparison, with imposter syndrome of if their content was good enough. All of these experiences were underpinned by a pressure to keep putting out content fearing becoming redundant, adapting their posts to become increasingly more performative and with less detailed information to meet the algorithm's requirements.

The psychologists exhibited anxiety at the variety of ethical issues they may encounter online, with great uncertainty over whether or not their behaviour was ethical. One of the most divisive topics covered was self‐disclosure and authenticity, with Harris and Robinson Kurpius (2014) conceding that social media has increased the potential for therapist self‐disclosure. Baier (2019) believe it is the psychologist's responsibility to ensure their behaviour on social media adheres to professional conduct and would not be detrimental to the therapeutic relationship if viewed by a client. This creates a pressure on the psychologist to analyse their every action online fearing unprofessional behaviour. Similarly to the participants in this study, Hynes et al. (2023) found marriage and family therapists may not self‐identify as influencers and thus not utilise reflexive decision‐making to consider the ethics of their social media use.

The psychologists experienced burnout in their roles in the NHS or third sector, so felt motivated to leave to protect their mental health (Ryan et al., 2019). Mental health care staff are at increased risk of burnout compared to other health care staff (Johnson et al., 2018). However, the psychologists were then faced with new risks to burnout working as a MHI, which are not as commonly considered or understood as the burnout risks from traditional psychology work. Clients may interpret their psychologist's profile as an indication of their increased accessibility and availability, with expectations that they can contact their psychologist for support whenever needed (Lehavot, 2009; Wardi‐Zonna et al., 2020). This places increased demands on the psychologist to always be checking their profile, increasing burnout risk.

Despite the risks to the psychologists' mental health noted above, every psychologist spoke of the positives of using Instagram as a MHI. The psychologists particularly valued the online community, and the unexpected friendship and camaraderie they had built with other MHIs. They believed Instagram created a space to engage their creativity and learn about psychological theories which could improve their practice. The psychologists spoke of how rewarding Instagram could be to work in line with their values, such as sharing social justice content, and to hear direct feedback of how they had helped others. All of these positives appeared to outweigh the negatives, as the psychologists opted to stay working as a MHI.

### Recommendations

Although guidance has been provided for therapist social media use with a personal presence (APA, 2021; BACP, 2023a; BPS, 2023; HCPC, 2024a), there is still ambiguity around how this guidance can be applied to an influencer presence (Smith et al., 2021). This study has identified key areas of an influencer presence which need further consideration and clarification within ethical guidance with recommendations provided below for how psychologists can implement these.

A key recommendation for ethical guidance is the need for supervision for professional use of social media. Supervision is beneficial on two levels: firstly, to support the psychologist with any ethical issues they may experience online; and secondly to care for the psychologists' mental health. Crucially, supervision checks the therapist's own well‐being and fitness to practise as they may not realise their proximity to burnout (Hawkins & Shohet, 2012). The BPS (2023) encourage psychologists to consider the disinhibition effect on social media for if they would share this information in a different context, such as a photo of themselves crying. This may be difficult to decide, as some psychologists interviewed wanted to model sharing emotions. Therefore, supervision can support psychologists with navigating the line between authentic self‐presentation on social media to increase engagement with posts and attract future clients, whilst still protecting their safety and professionalism.

A second recommendation regarding supervision is for supervisors to ongoingly educate themselves on how to use social media with different presences, and the various ethical issues which may arise to support their supervisees in minimising and mitigating risks. The APA (2021) recommend psychologists train their supervisees in ethical and professional uses of social media, yet this is at odds with the experiences shared in this study and other literature that supervisors may have less knowledge of social media than their supervisees and so cannot adequately support them (White & Hanley, 2023).

A third recommendation is for ethical guidance to stipulate clearer and firmer boundaries for professional social media use. People may assume MHIs are constantly available and able to support them in a crisis. Consequently, this study recommends MHIs communicate their boundaries through a pinned post or highlight. This should include that they can only provide signposting to other support services rather than directly managing risk or providing individualised support (Pretorius et al., 2019). Pretorius et al. (2022) reported more mental health professionals on Instagram provided disclaimers about the limitations of the support they could provide and signposted to crisis services, compared to professionals on TikTok. A further recommendation regarding boundaries is for psychologists to clarify they can only support individuals in one‐to‐one therapy if they have availability within their private practice, and reiterate that therapy would not take place via social media.

The fourth recommendation is for ethical guidance to advise psychologists to input boundaries around total time spent online with an influencer presence. The psychologists reported feeling exhausted after spending hours on Instagram each evening after work, supported by White and Chen‐Wilson (2023) reporting counsellors overuse of social media keeping their phones by their bed to maximise time online. Through conceptualising professional use of social media as a second job (irrespective of if they receive money from this work), this could help psychologists to implement boundaries and switch off at night. This study recommends having a separate professional account, so psychologists can interact with friends and family on their personal account without accidentally slipping into work. The BACP (2023b) advise psychologists to care for their well‐being when using social media with a personal presence, acknowledging the benefits of breaks as social media can be overwhelming. This emphasis on self‐care should be extended to MHIs, due to the extra pressures, such as burnout from additional work and receiving messages sharing trauma and risk.

A fifth recommendation is for ethical guidance to support psychologists with combining the roles of a psychologist and a businessperson to market their services on social media, acknowledging the potential contradictions between these identities. For example, influencers are encouraged to engage with followers through messages to promote products and to put passive products behind paywalls, which may feel incompatible with the boundaries and values of a psychologist. Psychologists should gain consent from clients to share anonymised testimonials, with discussion of the power dynamic and how the client would feel for others to see this. This study recommends psychologists consider the power their professional title holds, which could influence the public to purchase any products they promote. The psychologist should consider what types of products they could ethically promote through a sponsored post, if any, and if they have the competency to promote this. Whilst psychologists may be asked by the media to discuss issues outside of their area of expertise which could raise their public profile, they should only comment on issues within their competency.

A sixth recommendation is that psychologists use their Dr. title in their Instagram name and handle, and clearly state their protected title in their bio to indicate their competency gained from their professional training (HCPC, 2023). This title distinguishes the practitioner psychologist from others online sharing psychoeducation from lived experiences, or using unregulated titles which give no indication of level of training. Exceptions could be made for psychologists working in settings where knowledge of their identity could place them at risk, such as the participant in a forensic setting who chose an anonymised account. Psychologists still in professional training should clearly state they are a trainee, and not use protected titles until qualified. Psychologists should ideally create their own content, and if they outsource content creation this should be to individuals with mental health training. Psychologists should then review the content to take responsibility and help prevent misinformation being shared which the public would believe is from a credible source.

Concerningly, although people may try to use social media positively to access mental health information, the content they find may be stigmatising and actually deter accessing further support (Birnbaum et al., 2017). Therefore, a final recommendation is for ethical guidance to include how MHIs can create content which is not detrimental to public mental health. This study recommends that psychologists are considerate of their social media posts as a MHI taking into account that the information provided is drawing upon evidence‐based research that is within the psychologist's competency to discuss, and if possible providing references that are accessible to the public to read. This study advises that the information shared within the post is not oversimplified to a level that removes nuance and may lead to misinterpretation or misdiagnosis. Many of the psychologists in this study recommended sharing simple CBT resources as they were simple enough concepts to fit within the size constraints of a carousel or reel and could socialise an individual to the CBT model ahead of potentially receiving CBT within the NHS. Moreover, posts which contain highly emotive content, such as around sexual abuse, should contain trigger warnings and provide signposting to other services where individuals can receive tailored support. The psychologist may need to state the country within which they practice, to only provide signposting for services they are familiar with, as it would not be possible to provide knowledgeable signposting to support services in every country.

Awareness is needed that social media apps are constantly evolving, and so ethical guidance will need to be updated as new ethical issues arise. Consideration is needed within ethical guidance of how different social media apps may be used for different purposes, such as Instagram currently being used more for marketing therapeutic services compared to TikTok (Pretorius et al., 2022), in order to encompass the wide variety of ethical issues on social media.

### Strengths, limitations and areas for future research

This novel study is the first to qualitatively interview psychologists to understand their experiences of using Instagram as a MHI. Whilst many papers question ethical issues around psychologist use of personal social media accounts, this paper is novel through specifically focusing in on psychologist use of professional social media accounts as a MHI. The study is also the first to research the impact of using social media with an influencer presence on the psychologists' own mental health.

This study has provided a Western predominately white female view of psychologist social media use as a MHI, with the participants all within a 16‐year age bracket of 29–45. Although this is largely representative of the population of UK‐based psychologist MHIs on Instagram (and of psychologists in the UK including the primary researcher), more research is needed into the experiences of MHIs from other countries and with other diversity characteristics, such as age, race, and gender. Coincidentally, multiple participants focused their content on perinatal mental health, whilst only one participant (notably the only male) focused their Instagram on male mental health. Therefore, future research could explore if psychologists adapt their presentation of psychoeducation and how they engage with followers dependent on their target audience.

A recommendation for future research is to interview members of the public who engage with MHIs on social media. Multiple studies have questioned if people can distinguish between reputable mental health advice on social media, potentially assuming any guidance from a health professional must be a reliable source of psychoeducation (Feng & Campbell, 2011; McCashin & Murphy, 2023; Pretorius et al., 2019). Therefore, participants could discuss what they liked and disliked from MHI accounts, and how they identified reputable accounts to follow.

## CONCLUSION

This study has illustrated how social media use can be used positively by psychologists, during a time of increased psychological distress and systemic issues, with the provision of more comprehensive ethical guidelines, supervision, and training. Updated guidance specifically for a MHI presence can reduce psychologists' anxieties and potential risks around current ethical ambiguities. Psychologist motivations for working as a MHI appear layered and contradictory, with tensions between using social media for social media advocacy and marketing individual therapy services.

## AUTHOR CONTRIBUTIONS

**Ella White:** Conceptualization; investigation; writing – original draft; writing – review and editing; methodology; resources; formal analysis; visualization; project administration. **Terry Hanley:** Supervision; writing – review and editing.

## CONFLICT OF INTEREST STATEMENT

The authors declare no conflicts of interest.

## Data Availability

The data that support the findings of this study are available from the corresponding author upon reasonable request.

## APPENDIX A

### INTERVIEW SCHEDULE

Thank you for participating in the study. Taking part in this study is completely voluntary. You can request for your data to be withdrawn up to one week after the interview without giving a reason and your data will be destroyed. The interview will last approximately one hour, and you do not need to answer all of the questions if you do not want to.

Would you like to choose your own pseudonym?

Demographic info: age, gender, ethnicity.

Warm‐up questions contextualising the research:

1. What is your **job role** and **professional qualifications**?
2. How would you **describe** your social media platform?
3. What is your **own relationship** with social media like?
4. How do you **present yourself** on social media as a psychologist?
5. What **motivated** you to use your social media platform as a psychologist?
  *Prompts: stigma, changing role of practitioners, psychoeducation, social justice*

Questions aligned to the rationale exploring good practice:

6 How do you **balance your offline work** with your social media work?
7 How do you feel using social media as a psychologist **impacts your mental health**? *Prompts: burnout, resilience, trolling*
8 What are your **boundaries for separating your private life** from your social media presence as a psychologist?
  *Prompts: privacy, sharing personal photos and private information such as children and holidays*
9 What level of **knowledge and training** do you feel you need to share mental health information online?
  *Prompts: consideration of general public sharing mental health information compared to a professional, decisionson what content to share*
10 What would you **recommend other psychologists should do as good practice** when using social media as a mental health influencer?
11 Is there **anything else that you feel is relevant** that we have not discussed related to good practice?

Ok that is the end of the interview. Thank you so much for your participation today, and for sharing your experiences with me. Do you have any questions about the study?

I'll email you a debrief sheet with useful contacts in case todays interview has brought up any feelings for you. The sheet also has my email address and my supervisors email address, in case you have any further questions or want to withdraw your participation within the next week. With your consent, I will contact you in the future via email to discuss your views on the themes generated from my research (member checking) and later with the outcome of my research.

I'd also really appreciate if you could please share the study with anyone you think may be interested.

## APPENDIX B

Collapsed code map showing high interrelation between codes

Candidate themes map showing high interrelation between codes.
